# Supplementary figures and images for: Estimating person-specific neural correlates of mental rotation: A machine learning approach
Source: PLoS One. 2024 Jan 31;19(1):e0289094. doi: 10.1371/journal.pone.0289094 (PMC10830051; doi:10.1371/journal.pone.0289094)

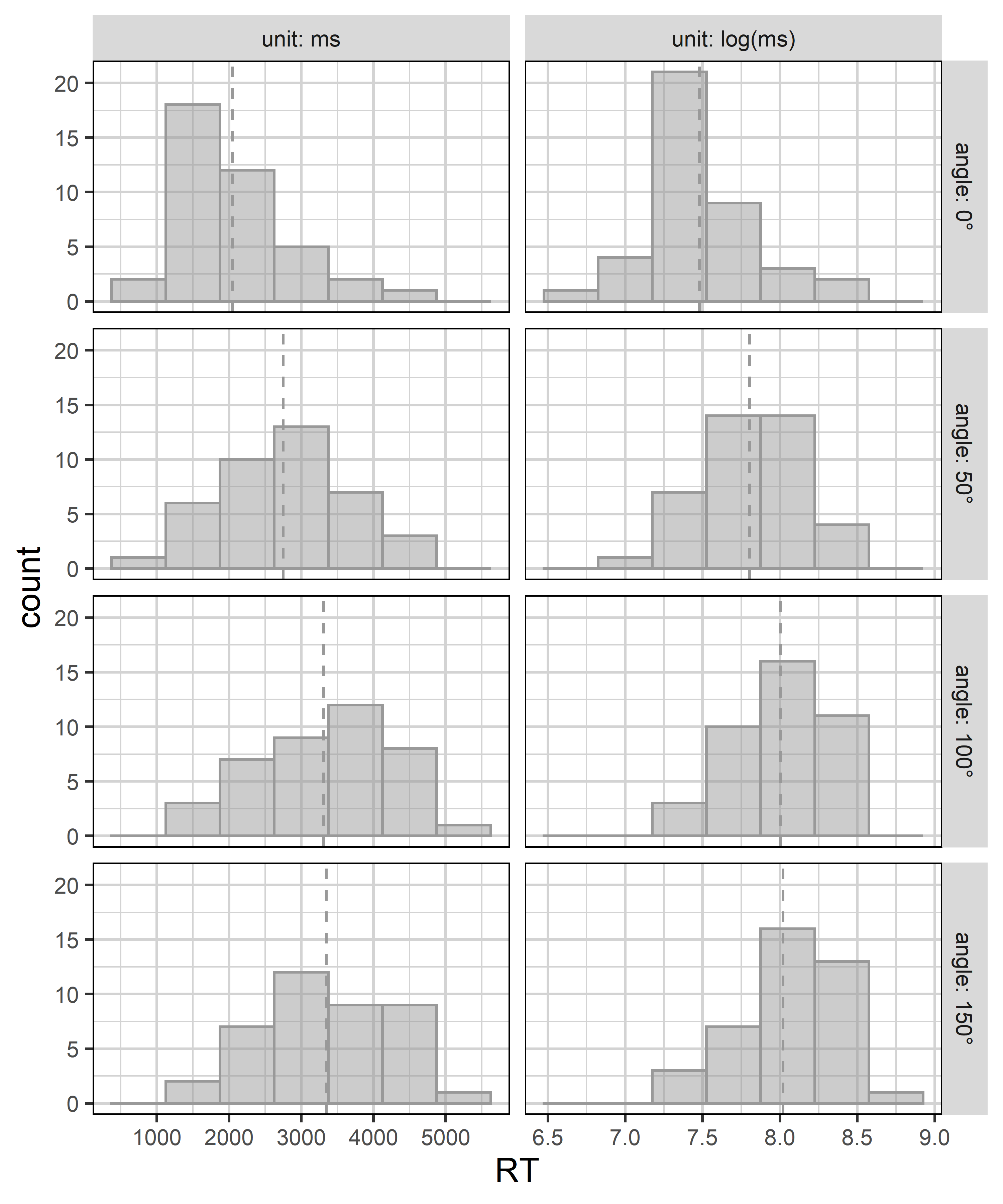

Supplement: S1 Fig — Histograms visualizing the distribution of participant-level reaction time (RT) averages per angular rotation (i.e., 0°, 50°, 100°, 150°) and unit (i.e., ms, log(ms)) included as labels in the training set. The dotted lines indicate the average RT per angular rotation and unit. (TIF) [file pone.0289094.s001.tif]
